# Safety of Pneumatic Dilation in Older Adults with Achalasia: An International Multicenter Cross-Sectional Study

**DOI:** 10.3390/jcm12206682

**Published:** 2023-10-23

**Authors:** Nir Bar, Christopher Vélez, Trisha S. Pasricha, Tamar Thurm, Dana Ben-Ami Shor, Roy Dekel, Yishai Ron, Kyle Staller, Braden Kuo

**Affiliations:** 1 Gastroenterology and Hepatology Department, Tel Aviv Medical Center, Tel Aviv Faculty of Medicine, Tel Aviv University, Tel Aviv 6423906, Israel; tamarth@tlvmc.gov.il (T.T.); danabe@tlvmc.gov.il (D.B.-A.S.); royd@tlvmc.gov.il (R.D.); yishair@tlvmc.gov.il (Y.R.); 2 Center for Neurointestinal Health, Division of Gastroenterology, Department of Medicine, Massachusetts General Hospital, Harvard Medical School, Boston, MA 02114, USA; cvelez@mgh.harvard.edu (C.V.); tpasricha@mgh.harvard.edu (T.S.P.); kstaller@mgh.harvard.edu (K.S.); bkuo@mgh.harvard.edu (B.K.)

**Keywords:** older adults, achalasia, pneumatic dilation, adverse events, safety

## Abstract

Background: Pneumatic dilation (PD) is an effective first line treatment option for many patients with achalasia. PD use may be limited in adults with achalasia who are older than 65 because of concern for adverse events (AE), and less efficacious therapies are often utilized. We explored the periprocedural safety profile of PD in older adults. Methods: An international real world cross-sectional study of patients undergoing PD between 2006–2020 in two tertiary centers. Thirty-day AEs were compared between older adults (65 and older) with achalasia and younger patients. Results: A total of 252 patients underwent 319 PDs. In 319 PDs, 18 (5.7%) complications occurred: 6 (1.9%) perforations and 12 (3.8%) emergency department referrals with benign (non-perforation) chest pain, of which 9 (2.8%) were hospitalized. No bleeding or death occurred within 30 days. Perforation rates were similar in both age groups and across achalasia subtypes. Advanced age was protective of benign chest pain complications in univariate analysis, and the limited number of AEs precluded multivariable analysis. Conclusions: The safety of PD in older adults is at least comparable to that of younger patients and should be offered as an option for definitive therapy for older patients with achalasia. Our results may affect informed consent discussions.

## 1. Background 

Achalasia is a neurodegenerative disease resulting in impaired lower esophageal sphincter (LES) relaxation and esophageal food retention predisposing patients to aspiration-related complications. Standard of care invariably includes LES disruption, achieved by one of three modalities: serial pneumatic dilations (PDs) which are performed during upper endoscopy, or endoscopic or surgical myotomy [1,2,3]. Notably, in recent years, peroral endoscopic myotomy, originating from Japan, has gained increasing favor worldwide as an efficacious and safe therapeutic modality for achalasia [4]. Although it is gaining popularity in the West, its prevalence remains limited, and it is still not universally available [5]. Laparoscopic Heller myotomy, on the other hand, is an available treatment option, which is often combined with partial fundoplication to reduce gastroesophageal reflux complications. Despite being more available than endoscopic myotomy, its use is limited by patient comorbidities like other surgeries [6]. For many years, PD has been the mainstay of achalasia treatment, and remains so where endoscopic myotomy is unavailable [7]. PD is performed under sedation during an esophagogastroduodenoscopy (EGD) with a balloon inflated with air at the LES. The procedure time is shorter than other techniques, with most patients being discharged on the same day. PD is performed in a sequential fashion, wherein the endoscopist incrementally increases the balloon diameter in each successive procedure to mitigate the risk of adverse events (AEs) [8].

With the aging population, older adults with achalasia may increasingly need treatment. We know older age is associated with better PD efficacy, with previous studies and meta-analyses showing that older age predicts a higher likelihood of PD success. Studies show both short and long term success, with fewer PDs required [7,9,10,11,12,13]. Nonetheless, real-world data indicate that patients who undergo PD, especially in non-university-based practices, are considerably younger than those treated with other less effective endoscopic therapies [14,15]. 

Presumably, the management of older adults is affected by the perception of increased risk. The few studies which include age-related safety data have contradictory results. Some note higher AE rates, including bleeding, esophageal hematoma, aspiration pneumonia, chest pain, and fever [2,3,16,17,18,19], while others do not [7]. We aimed to explore the safety profile of PD in older adults with achalasia. 

## 2. Methods 

This was an international multicenter retrospective cross-sectional study. We included all consecutive PDs in adult patients with achalasia at Tel Aviv Medical Center (TLVMC) in Israel and Massachusetts General Hospital (MGH) in Boston, MA, USA, from November 2006 to October 2020. For each case, we recorded demographic data with age at time of PD, type of achalasia (according to Chicago classification 3.0. [20] when possible), diameter of PD, and periprocedural adverse events (AE). Achalasia types were reported when the manometry was performed after the adaptation of the Chicago classification 3.0 classification, which is identical to the achalasia definitions in the 4.0 iteration currently being used [21]. Patients with missing data were excluded from the study.

PDs were performed per institutional standard. Rigiflex balloon dilators, (Boston Scientific, Marlborough, MA, USA) were used by both centers during the study period. Both centers performed PDs under fluoroscopic guidance. 

At TLVMC, the LES was marked with a submucosal injection of contrast. PD occurred in a stepwise approach over time using a 30 mm, 35 mm, and a third dilation of either 35 mm or 40 mm balloon, depending on endoscopist preference. Balloons were inflated to 9 PSI for either a 60 s period or two consecutive 30 s each time with readjustment of the balloon when it was deflated. The esophagus was surveyed endoscopically for perforations and/or esophageal tears after balloon withdrawal. At MGH, the LES was located endoscopically and marked with a radiopaque marker externally (i.e., straight mosquito forceps attached to the patient gown) and by finding of a balloon waist. Initially a 30 mm balloon was used, inflated once for 60 s to a pressure of 20 PSI. Similarly, subsequent PDs were performed in a stepwise approach of increasing balloon diameter. In both centers, advancing to larger balloons relied on both clinical response and barium swallow or functional luminal impedance planimetry studies.

We recorded age at time of PD (older adults were defined as ≥65 years), sex, achalasia type, PD diameter, and periprocedural adverse events (AE, defined as occurring within 30 days of the procedure). AEs of interest included esophageal perforations, bleeding, PD-associated Emergency Department (ED) referrals, and hospitalizations. Chest pain severe enough to require an ED evaluation was termed “benign chest pain” after ruling out perforation by computed tomography. 

In Israel, each hospital’s medical records are connected to the ministry of health database; a centralized database, allowing access to information about mortality and date of death for the entire population. In the TLVMC cohort, survival status and date of death data were collected as well. While survival status was also recorded in the MGH cohort, mortality data were not linked to the MGH database, so these patients were not included in the mortality analysis.

Our study included patients with achalasia of unknown type if they underwent PD before the Chicago classification 3.0 was published, or when the catheter could not traverse the LES, but achalasia was confirmed with a normal endoscopy and a characteristic imaging study (e.g., a barium esophagram with a wide lumen esophagus ending with a smooth “bird’s beak” shape LES).

Data were examined for normal distribution by using Q–Q plots and normalcy tests. Data are presented as mean ± standard deviation. Categorical parameters are presented as n (%). *t*-test and Chi square tests were used to compare groups. SPSS was used for all statistical analysis (IBM SPSS statistics version 22.0, 2013 IBM corp., Armonk, NY, USA). When discussing patient characteristics, results are reported per patient (*n* = 252). When discussing AEs, results are reported per procedure (*n* = 319). For each procedure, the patient age was calculated at the time of the PD.

This study was approved by each center’s respective institutional review board. For the STrengthening the Reporting of OBservational studies in Epidemiology (STROBE) checklist, see Appendix A.

## 3. Results

The cohort included 252 patients (197 from TLVMC, 55 from MGH) who underwent 319 PDs between November 2006 and October 2020. Age, sex, achalasia type, and number of PDs were similar between centers (Table 1). 

There were 114 (45.2%) older adults. The older adult and younger than 65 groups were similar in sex and achalasia type distribution (Table 2). Eighteen (5.6%) PDs were followed by AEs: six (1.9%) perforations and twelve (3.8%) benign chest pain events (starting within the first 24 h). Nine (2.8%) patients were hospitalized for benign chest pain. All nine patients were discharged promptly (up to 48 h). There was no 30-day mortality or bleeding. All AEs occurred at TLVMC. 

**Figure 1:**
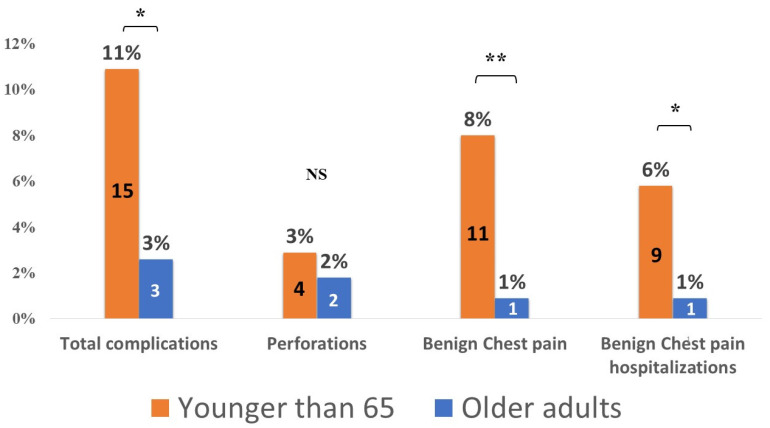
Complications across age groups. *—*p* < 0.05, **—*p* < 0.01, NS—non-significant.

Older adults had significantly fewer total AEs. Total periprocedural complications in the older adults group compared to the younger group were 3 (2.2%) vs. 15 (8.4%), *p* = 0.017. (Table 2 and Figure 1). The difference was driven by fewer benign chest pain episodes, while the perforation rates were similar. Benign chest pain occurred in 1 (0.7%) patient in the older adults group compared to 11 (6.1%) in the younger group, *p* = 0.012. The benign-chest-pain-related hospitalizations rate was also lower with 1 (0.7%) hospitalization in the older adults compared to 8 (4.5%) hospitalizations in the younger group, *p* = 0.045. Esophageal perforation rates, however, was similar between groups: two (1.4%) in the older compared to four (2.2%) in the younger group, *p* = 0.699.

We used univariate logistic regression to assess the effect of age on AEs. For each 5-year increase in age the OR (95% CI) for total AEs was 0.85 (95% CI 0.74–0.97) and for chest pain was 0.78 (95% CI 0.66–0.92). No association was found with perforation (OR 1.01, 95% CI 0.8–1.28). Because of the low number of AEs, multivariate analyses could not be performed. 

Of note, all perforations occurred during the first PD using a 30 mm balloon other than one in the younger group which was the second PD using a 35 mm balloon. One of the two perforations occurring in the older adults group resulted in death from sepsis 3 months after the perforation was sealed using an over the scope clip. This was the patient’s first PD with a 30-mm balloon. The other patient older than 65 who experienced a perforation was treated conservatively. Of the four patients younger than 65 who experienced a perforation, three were managed conservatively, and one underwent surgical intervention and was subsequently discharged.

## 4. Discussion

In this multicenter cohort with more than 300 PDs, we found a lower rate of periprocedural AEs in older adults with achalasia compared to younger patients. Perforation rates were similar in both groups, but post-procedural chest pain was more common in patients younger than 65. There was no 30-day mortality in either group, though one patient from the older adults group died after a long hospitalization following a perforation. 

PD is widely used in the management of achalasia and carries an inherent risk of AEs. Compared to younger patients, older adults are generally known to have more comorbidities and higher rates of AEs during interventions such as colonoscopies [22] and noncardiac surgeries [23]. Our cohort perforation rate for both the older than 65 and younger groups falls within the previously described 0–5% range in the systematic review and meta analysis by Van Hoeij et al. [24]. The effect of advanced age on procedural safety is of increasing interest, as data are inconclusive. 

Similar to our results, another large retrospective study demonstrated a perforation rate of 1.3% for PD without increased risk with age and no deaths (though no age specific estimates were reported) [25]. Conversely, a randomized controlled trial demonstrated increased risk of perforation in older patients as those with perforation were older than those without (61 vs. 36 year-olds, *p* = 0.003). No deaths were reported. However, the PD protocol in this study had to be amended following the recruitment of 13 patients because of a high perforation rate (4/13), which might explain the discrepancy between our results [19]. This study also underscores the importance of carful patient selection and the use of graded balloon diameter in successive procedures. In a retrospective study of 237 patients, 7 (1.3%) esophageal perforations were observed. An additional 8 (1.6%) patients had asymptomatic mucosal tears, hematomas, and fever [16]. Patients with complications were older than those without (mean age −68.5 ± 15 compared to 56.4 ± 20, *p* < 0.05, though for perforations alone the difference was not statistically significant). Two patients older than 91 died following a perforation. In a recent single center study in Turkey examining the safety and efficacy of PD by Tenlik et al., the perforation rates were similar in patients above and under 65, (0.7% in both groups) [26].

In a national database study, 1.6% of 4748 PDs were complicated by perforations but not associated with age. Patients aged 66–77 and >77 had a 30-day mortality of 1.3% and 5.1%, respectively. The adjusted OR for 30-day mortality was 4.55 (2–10.38) for ages 66–77 and 9.78 (4.33–22.06) for age > 77 (compared to the 18–38 quintile). Of note, the post-perforation-mortality rate was 6/77 (<8%), but the other 83/89 patients died from non-perforation-related reasons within 30 days of PD. A Charlson comorbidity score > 4, rather than perforation, was independently associated with increased mortality [7]. The increased mortality for this study relative to ours is striking. This may be explained by patient selection, center volume (tertiary centers vs variable care levels in the database study), and PD technique. 

Since PD efficacy in older adults is as good if not better than in younger patients [7,27], our study suggests it is also a safe management option and should probably be centralized to specializing centers. Definitive achalasia treatment which improves esophageal emptying could help reduce aspiration complication which may be important to patients older than 65.

An interesting finding in our study was the association of post-procedural chest pain with young age. To our knowledge, this association has not been previously reported. Benign chest pain, as a complication unrelated to perforation, is generally not widely reported. Post-procedural benign chest pain was documented in 15–16.2% of patients who underwent PD, but these were not stratified according to age [12,13,18] and might be explained by reduced esophageal sensitivity in older individuals. Lasch et al. show that healthy individuals who were older than 65 have previously tolerated higher inflation volumes of an intraesophageal balloon compared to younger controls, implying diminished visceral pain perception [28]. Furthermore, in another study by Anggianasah et al., older patients with gastroesophageal reflux disease symptoms were found to have higher levels of esophageal acid exposure compared to younger patients, despite having similar symptoms severity scores [29].

Our findings, which confirm the overall safety of PD in adult patients, coupled with the potential for higher efficacy, offer valuable insights for guiding patient management strategies. We continue to advocate for a less invasive approach, particularly in older patients burdened by multiple comorbidities, where the importance of thoughtful patient selection cannot be overstated. For such individuals, the injection of botulinum toxin into the LES remains a prudent choice.

However, older adults who are good candidates for surgical interventions may pursue more invasive LES-directed therapies. In situations where peroral endoscopic myotomy is not an available option, PD could continue being the treatment of preference, especially for patients who wish to avoid surgery or hospitalizations. Our research provides patients and physicians with important information regarding the expected rate of adverse events, particularly noting an elevated incidence of chest pain among younger patients, while the rate of perforation remains comparably low. In adopting a collaborative decision-making model, these insights are relevant within the context of the informed consent process. They serve as a valuable resource for ensuring that patients are thoroughly informed about their treatment approach.

We recognize limitations of this work, specifically differing procedural protocols between centers that may affect AE rates. That said, having two centers increases the overall generalizability of our findings. Selection bias may also exist, where older adults selected for PD may have been intrinsically healthier or with lower comorbidities than patients in whom the procedure was not performed, thus excluding patients with more comorbidities or perhaps longstanding more advanced achalasia from our study. Furthermore, we cannot rule out specific providers adjusting the protocol to older patients. We would argue, however, that this reaffirms the importance of careful patient selection so as to not deny an effective treatment to older individuals. 

In conclusion, we demonstrate the overall safety of PD across the age spectrum, which may have important implications for the large number of achalasia patients where access to endoscopic or surgical myotomy is still limited. Moreover, our data suggests that older patients can still receive definitive achalasia therapy rather than the less effective therapies frequently offered instead.

## Figures and Tables

**Table 1:** Patients baseline characteristics in each center.

	Total Cohort (*n* = 252)	TLVMC (*n* = 197)	MGH (*n* = 55)
Age	61.3 ± 17.4 Range: 18–97.9	60.7 ± 17 Range: 19.1–97.9	63.4 ± 18.8 Range 18–96.3
Adults older than 65	114 (45.2)	85 (43.1)	29 (52.7)
Adults older than 70	86 (33.6)	63 (32)	23 (41.8)
Adults older than 75	53 (20.7)	38 (19.3)	15 (27.3)
Female adults	116 (45.3)	86 (43.8)	28 (50.9)
Achalasia type ^a^	N = 124	N = 95	N = 29
Type 1	18 (14.5)	15 (15.8)	3 (10.3)
Type 2	95 (76.6)	70 (73.7)	25 (86.2)
Type 3	11 (8.9)	10 (10.5)	1 (3.4)
Number of PDs			
1	252 (78.9)	197 (77.2)	55 (85.9)
2	58 (18.2)	49 (19.3)	9 (14.1)
3	9 (2.8)	9 (3.5)	

^a^—Chicago classification 3.0 was available for 124 patients. Values are per patient and presented as mean ± SD or *n* (%) as appropriate.

**Table 2:** Characteristics and adverse events in older adults compared to the younger age group.

	Younger than 65 (*n* = 138)	Older Adults (>65) (*n* = 114)
Age ***	49 ± 12.9	76 ± 7.8
Female	61 (44.2)	53 (46.5)
Center—n (% within center)		
Tel Aviv Medical Center	112 (56.9)	85 (43.1)
Massachusetts General Hospital	26 (47.3)	29 (52.7)
Achalasia type (out of 124 typable cases)	N = 52	N = 72
Type 1	6 (11.5)	12 (16.7)
Type 2	41 (78.8)	54 (75)
Type 3	5 (9.6)	6 (8.5)
Adverse events		
All adverse events *^a^	15 (8.4)/(10.9)	3 (2.2)/(2.6)
Esophageal perforation ^a^	4 (2.2)/(2.9)	2 (1.4)/(1.8)
Benign chest pain **^a^	11 (6.1)/(8)	1 (0.7)/(0.9)
Death within 30 days of PD	0	0

^a^—adverse events are presented as *n* (% of pneumatic dilations)/(% of total patients). Continuous data were compared with *t*-test, and categorical were compared with Chi-square test. *—*p* < 0.05, **—*p* < 0.01, ***—*p* < 0.001. Statistical analysis was performed per patient. Values are per patient unless otherwise stated and presented as mean ± SD or *n* (%) as appropriate.

## Data Availability

The data presented in this study are available on request from the corresponding author. The data are not publicly available due to data confidentiality.

## Appendix A

**Table A1:** STROBE Statement—checklist of items that should be included in reports of observational studies.

	Item No.	Recommendation	Page No.	Relevant Text from Manuscript
Title and abstract	1	(a) Indicate the study’s design with a commonly used term in the title or the abstract	1	International Multicenter Cross-Sectional Study
		(b) Provide in the abstract an informative and balanced summary of what was performed and what was found	4	
Introduction				
Background/rationale	2	Explain the scientific background and rationale for the investigation being reported	5	
Objectives	3	State specific objectives, including any prespecified hypotheses	5	“We aimed…”
Methods				
Study design	4	Present key elements of study design early in the paper	5	“This was an international…”
Setting	5	Describe the setting, locations, and relevant dates, including periods of recruitment, exposure, follow-up, and data collection	5	“This was an international…”
Participants	6	(a) Cohort study—give the eligibility criteria, and the sources and methods of selection of participants. Describe methods of follow-up Case-control study—give the eligibility criteria, and the sources and methods of case ascertainment and control selection. Give the rationale for the choice of cases and controls Cross-sectional study—give the eligibility criteria, and the sources and methods of selection of participants	5	“We included all…”
		(b) Cohort study—for matched studies, give matching criteria and number of exposed and unexposed Case-control study—for matched studies, give matching criteria and the number of controls per case		
Variables	7	Clearly define all outcomes, exposures, predictors, potential confounders, and effect modifiers. Give diagnostic criteria, if applicable	6	“We recorded…”
Data sources/measurement	8 *	For each variable of interest, give sources of data and details of methods of assessment (measurement). Describe comparability of assessment methods if there is more than one group	*6*	”at TLVMC, the LES…”
Bias	9	Describe any efforts to address potential sources of bias		
Study size	10	Explain how the study size was arrived at	5	“We included all…”
Quantitative variables	11	Explain how quantitative variables were handled in the analyses. If applicable, describe which groupings were chosen and why		
Statistical methods	12	(a) Describe all statistical methods, including those used to control for confounding	7	“Data were examined…”
		(b) Describe any methods used to examine subgroups and interactions		
		(c) Explain how missing data were addressed	5	“Patients with missing…”
		(d) Cohort study—if applicable, explain how loss to follow-up was addressed Case-control study—if applicable, explain how matching of cases and controls was addressed Cross-sectional study—if applicable, describe analytical methods taking account of sampling strategy		
		(e) Describe any sensitivity analyses		
Results				
Participants	13 *	(a) Report numbers of individuals at each stage of study—e.g., numbers potentially eligible, examined for eligibility, confirmed eligible, included in the study, completing follow-up, and analysed	7	“The cohort included 252 patients…”
		(b) Give reasons for non-participation at each stage		
		(c) Consider use of a flow diagram		
Descriptive data	14 *	(a) Give characteristics of study participants (e.g., demographic, clinical, social) and information on exposures and potential confounders	14	See Table 1 and Table 2
		(b) Indicate number of participants with missing data for each variable of interest		
		(c) Cohort study—summarise follow-up time (e.g., average and total amount)		
Outcome data	15 *	Cohort study—report numbers of outcome events or summary measures over time		
		Case-control study—report numbers in each exposure category, or summary measures of exposure		
		Cross-sectional study—report numbers of outcome events or summary measures	14	See Table 1
Main results	16	(a) Give unadjusted estimates and, if applicable, confounder-adjusted estimates and their precision (e.g., 95% confidence interval). Make clear which confounders were adjusted for and why they were included	7	“We used univariate logistic regression…”
		(b) Report category boundaries when continuous variables were categorized	8	“In this multicenter cohort with more…”
		(c) If relevant, consider translating estimates of relative risk into absolute risk for a meaningful time period		
Other analyses	17	Report other analyses performed—e.g., analyses of subgroups and interactions, and sensitivity analyses		
Discussion				
Key results	18	Summarise key results with reference to study objectives	8	“we found…”
Limitations	19	Discuss limitations of the study, taking into account sources of potential bias or imprecision. Discuss both direction and magnitude of any potential bias	9	“We recognize limitations of this work…”
Interpretation	20	Give a cautious overall interpretation of results considering objectives, limitations, multiplicity of analyses, results from similar studies, and other relevant evidence	9	“In conclusion…”
Generalisability	21	Discuss the generalisability (external validity) of the study results	9	“In conclusion…”:
Other information				
Funding	22	Give the source of funding and the role of the funders for the present study and, if applicable, for the original study on which the present article is based	10	See conflict of interest, under acknowledgments

* Give information separately for cases and controls in case–control studies and, if applicable, for exposed and unexposed groups in cohort and cross-sectional studies. Note: An Explanation and Elaboration article discusses each checklist item and gives methodological background and published examples of transparent reporting. The STROBE checklist is best used in conjunction with this article (freely available on the Web sites of PLoS Medicine at http://www.plosmedicine.org/ (accessed on 6 June 2023), Annals of Internal Medicine at http://www.annals.org/ (accessed on 6 June 2023), and Epidemiology at http://www.epidem.com/ (accessed on 6 June 2023)). Information on the STROBE Initiative is available at www.strobe-statement.org (accessed on 6 June 2023).
